# Ambient Lights Influence Perception and Decision-Making

**DOI:** 10.3389/fpsyg.2018.02685

**Published:** 2019-01-09

**Authors:** Sichao Song, Seiji Yamada

**Affiliations:** ^1^Department of Informatics, The Graduate University for Advanced Studies (SOKENDAI), Tokyo, Japan; ^2^Digital Content and Media Sciences Research Division, National Institute of Informatics, Tokyo, Japan

**Keywords:** ambient light systems, expressive lights, human-machine interaction, peripheral display, affective computing

## Abstract

Today's computers are becoming ever more versatile. They are used in various applications, such as for education, entertainment, and information services. In other words, computers are often required to not only inform users of information but also communicate with them socially. Previous studies explored the design of ambient light displays and suggested that such systems can convey information to people in the periphery of their attention without distracting them from their primary work. However, they mainly focused on using ambient lights to convey certain information. It is still unclear whether and how the lights can influence people's perception and decision-making. To explore this, we performed three experiments using a ping-pong game, Ultimatum game, and Give-Some game, in which we attached an LED strip to the front-bottom of a computer monitor and had it display a set of light expressions. Our evaluation of the results suggested that expressive lights do affect human perception and decision-making. Participants liked and anthropomorphized the computer more when it displayed light animations. Particularly, they perceived the computer as positive and friendlier when it displayed green and low intensity light animation, while red and high intensity light animation was perceived as negative and more hostile. They consequently behaved with more tolerance and cooperation to the computer when it was positive compared with when it was negative. The findings can open up possibilities for the design of ambient light systems for various applications where human-machine interaction is needed.

## 1. Introduction

Electronic devices such as computers are widely used in our daily lives, either personally or publicly. They are used in various applications such as education, entertainment, and information services. In all cases, it is important for such devices to guarantee their users a pleasant and natural interaction experience. Such an objective has become an important research topic in human-computer interaction (HCI) and human-machine interaction (HMI) in general.

Many factors are related to this goal. Among them, anthropomorphism has been considered as one key factor for interaction design as it can influence a user's perception of a device substantially. According to Reeves and Nass (1996), people interact with new media in much the same way as they interact with other people. Moreover, Tremoulet and Feldman (2000) and Fussell et al. (2008) well-demonstrated the intrinsic mechanism of humans to anthropomorphize objects. For this reason, various studies tried to reach a more natural interaction design by using anthropomorphism methods such as adding human-like eyes and body parts to a device (Osawa and Imai, 2013) or providing human-like body movements (Hoffman et al., 2015).

Unfortunately, these methods are not applicable to many currently-in-use devices such as personal computers as most PCs at present use a keyboard, a mouse, and/or a touchpad as input modalities and a display (monitor) and/or a speaker as output modalities. It can be complex or even impractical to apply human-like design methods to such PCs. Thus, it is important to investigate new methods that can improve a user's interaction experience while being simple and adequate to apply.

To address this problem, we probe an alternative modality: expressive light. Light, as an interaction modality, has been widely studied in different fields. Many previous studies in fields such as psychology, HCI, and human-robot interaction (HRI) have investigated the effect of light and color on human perception. For instance, a number of researchers used expressive lights for their systems to either express affect (Snyder et al., 2015; Sokolova and Fernández-Caballero, 2015; Song and Yamada, 2017a) or convey certain information (Harrison et al., 2012; Szafir et al., 2015; Baraka and Veloso, 2018); a handful of papers discussed affective modulation using light and color (Picard, 2010; Sokolova and Fernández-Caballero, 2015).

Particularly with regard to HRI related studies, expressive lights have been considered as effective dynamic vision cues for appearance-constrained robots to communicate internal states and intent. Similar to a computer, such robots are neither anthropomorphic nor zoomorphic. The lack of expressiveness makes these robots' behaviors hard for people to understand. Therefore, HRI researchers explored the use of expressive lights in various contexts to indicate internal states (Baraka and Veloso, 2018), communicate intent (Szafir et al., 2015), and express emotion (Feldmaier et al., 2016).

In addition, expressive lights can be seen as a calming technology (Weiser and Brown, 1996). Systems that use light to convey information on the periphery of human vision are defined as ambient light systems (Matviienko et al., 2015). Users of such systems can perceive information encoded in the lights while maintaining focus on main tasks. Basically, it is suggested that expressive lights can be used to convey the information of four classes: progress, status, spatial, and notification (Matviienko et al., 2015). In particular, Müller et al. (2015) designed an ambient light display system named “Lighten Up.” They built a four-side frame equipped with individually controllable LEDs and mounted it to the back of a computer monitor. Further, they explored the design space with 42 light patterns and found that users prefer their ambient light system over an on-screen display.

Despite the promising results, previous work mainly focused on informing users of certain information, e.g., progress on tasks or notifications. It is still unclear whether and how expressive lights can influence people's perception, behavior, and decision-making. This is important as today's computers are becoming more versatile in various applications, such as for entertainment, education, business, and even social interactions. As a result, ambient light display systems used for different applications may have the potential to affect their users' psychological functioning and behaviors, either explicitly or implicitly. This will push forward the design of the systems toward a more ambitious goal: interacting with users.

**Figure 1:**
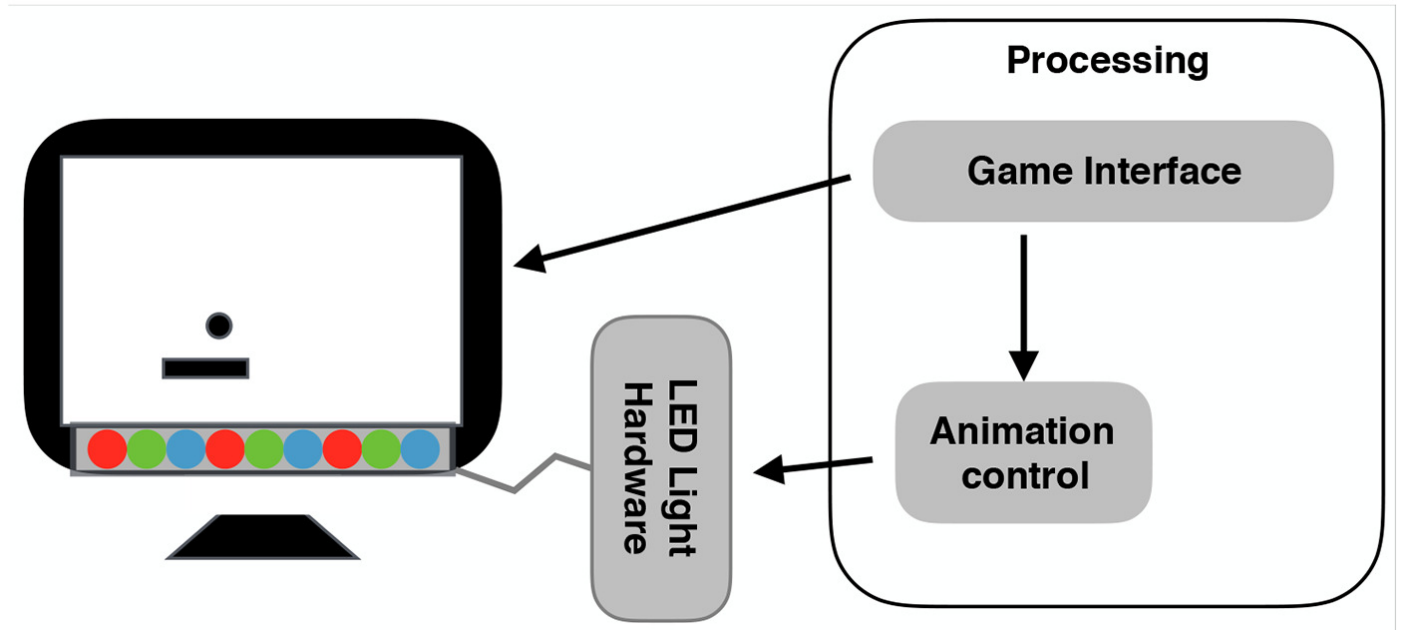
System overview.

To explore this, in this work, we attached a programmable LED strip to the front-bottom of a monitor (Song and Yamada, 2017b). On the basis of previous work (Yamada et al., 2013), we assumed that such a monitor placement is within a user's peripheral visual field and thus will not distract him or her. Figure 1 shows an overview of our system. We worked through a structured process to investigate our research question: whether and how expressive lights can affect people's perception and behavior toward a computer.

We divided our approach into two parts. In study 1, we developed a PingPong game for carrying out an experiment. We designed a set of LED light animations for various events that happen during the game, e.g., the racket hitting the ball, the racket hitting the walls, and game over. We collected experiment data by using a post-game questionnaire and game log data. The goal of this study was to observe whether adding expressive lights to game playing can influence people's attitude and performance of the game. Moreover, we were interested in whether such lights can further impact people's perception of the computer itself. Results of study 1 was reported in Song and Yamada (2017b).

To further investigate effects of expressive lights on people's perception and decision-making, we performed a series of two more studies. In study 2, we introduced two games, the Ultimatum game and Give-Some game. Differing from the PingPong game, these two games require people to make economic decisions and thus can be used to measure human altruistic behavior (Güth and Tietz, 1990). During each game, the LED strip displayed pre-designed light animations together with the proposals offered by the computer. We collected experiment data by using post-game questionnaires and game logs. The goal of this study was to explore whether and how expressive lights can influence people's decision making toward the computer.

Findings from both studies together will contribute to deeper understanding of the effects that expressive lights have on humans and further open up possibilities for the design of ambient light systems for various applications.

## 2. LED Strip Light Animation

Baraka and Veloso (2018) used expressive lights to reveal their mobile service robot's states. As we used the same LED strip, an Adafruit NeoPixel strip with 144 programmable LED pixels per meter, we adapted the light animation pattern definitions from their work. In order to fit the width restriction of our monitor, we used a half meter of the LED strip (72 pixels) (Song and Yamada, 2017b).

We define an animation A(t) of 72 pixels as a time-varying 72 × 3 matrix of color intensities:

(1) $$\begin{array}{r} A\left(t\right)=\left(\begin{array}{ccc} i_{1r} & i_{1g} & i_{1b}\\ i_{2r} & i_{2g} & i_{2b}\\ \vdots & \vdots & \vdots \\ i_{72r} & i_{72g} & i_{72b} \end{array}\right), \end{array}$$

where the rows represent the indices of pixels and the columns represent the three color channels *r*, *g*, and *b*. The intensity values are the values of the three channels, respectively:

(2) $$\begin{array}{r} \forall :0\leq i_{jc_{k}}\leq 255j=1,\cdots \;,72;c_{k}=r,g,b \end{array}$$

## 3. Study 1

### 3.1. Methods

#### 3.1.1. Experiment Design

**Figure 2:**
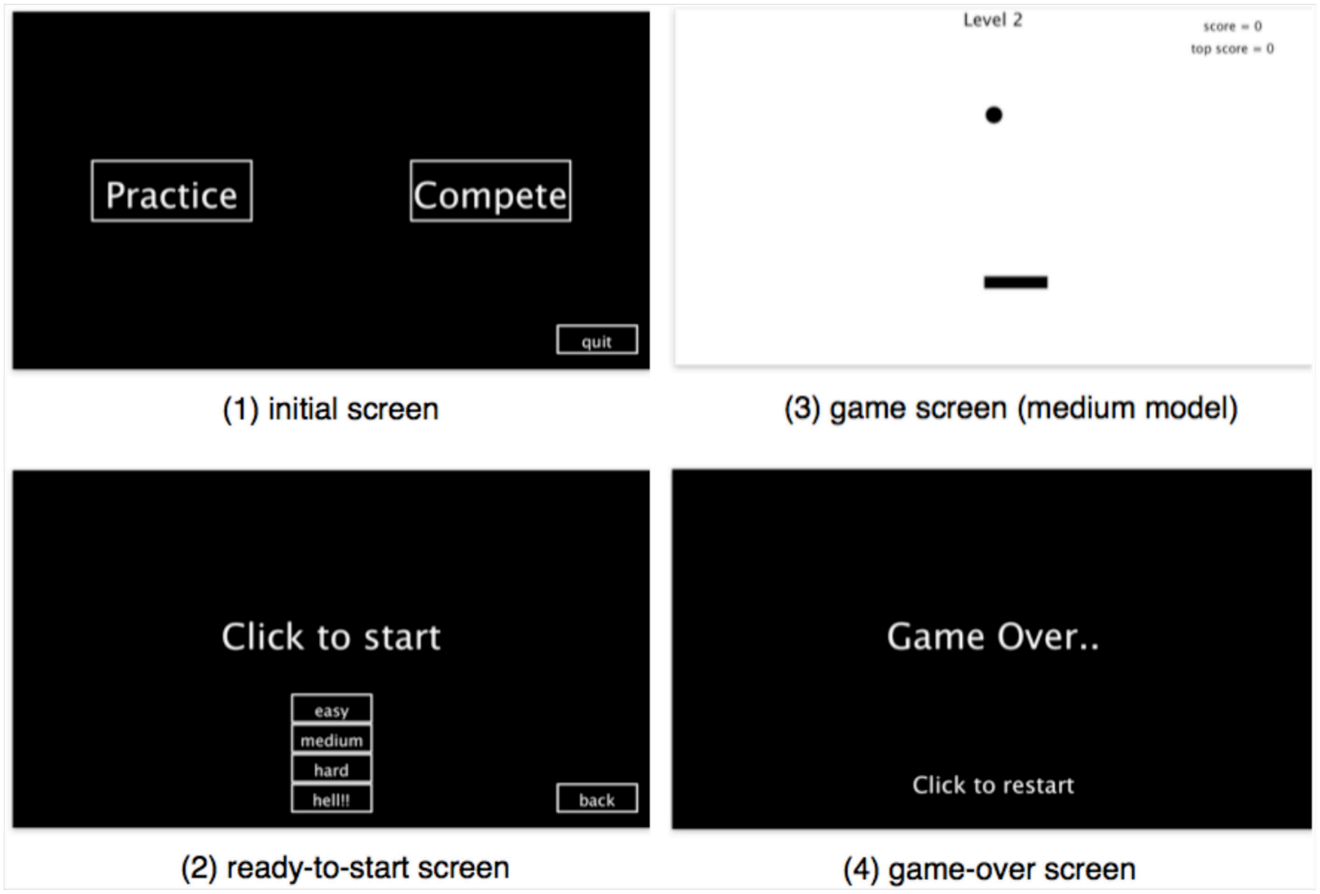
Four screen shots of ping pong game.

We developed the ping-pong game using Processing. Figure 2 shows different game screens: an initial screen, ready-to-start screen, in-game screen, and game-over screen. We set the goal of the game as to bounce the ball (moving the racket by mouse) to reach a high score. Four difficulty levels were designed by setting different racket lengths and horizontal forces on the ball when it hits the racket to meet the participants' different gaming abilities. Basically, we designed different scoring metrics with regard to the difficulty levels, where a player get 1 point each time the ball hits the racket in the easy mode, 2 points in the medium mode, 5 in the hard mode, and 10 in the hell mode. We observed five events in the ping-pong game: waiting for game to start, ball hits racket, ball hits wall, playing, and game over. Each event was coded uniquely, and the corresponding code was sent to an Arduino board to control the LED strip to display event-triggered light animations on-the-fly.

**Figure 3:**
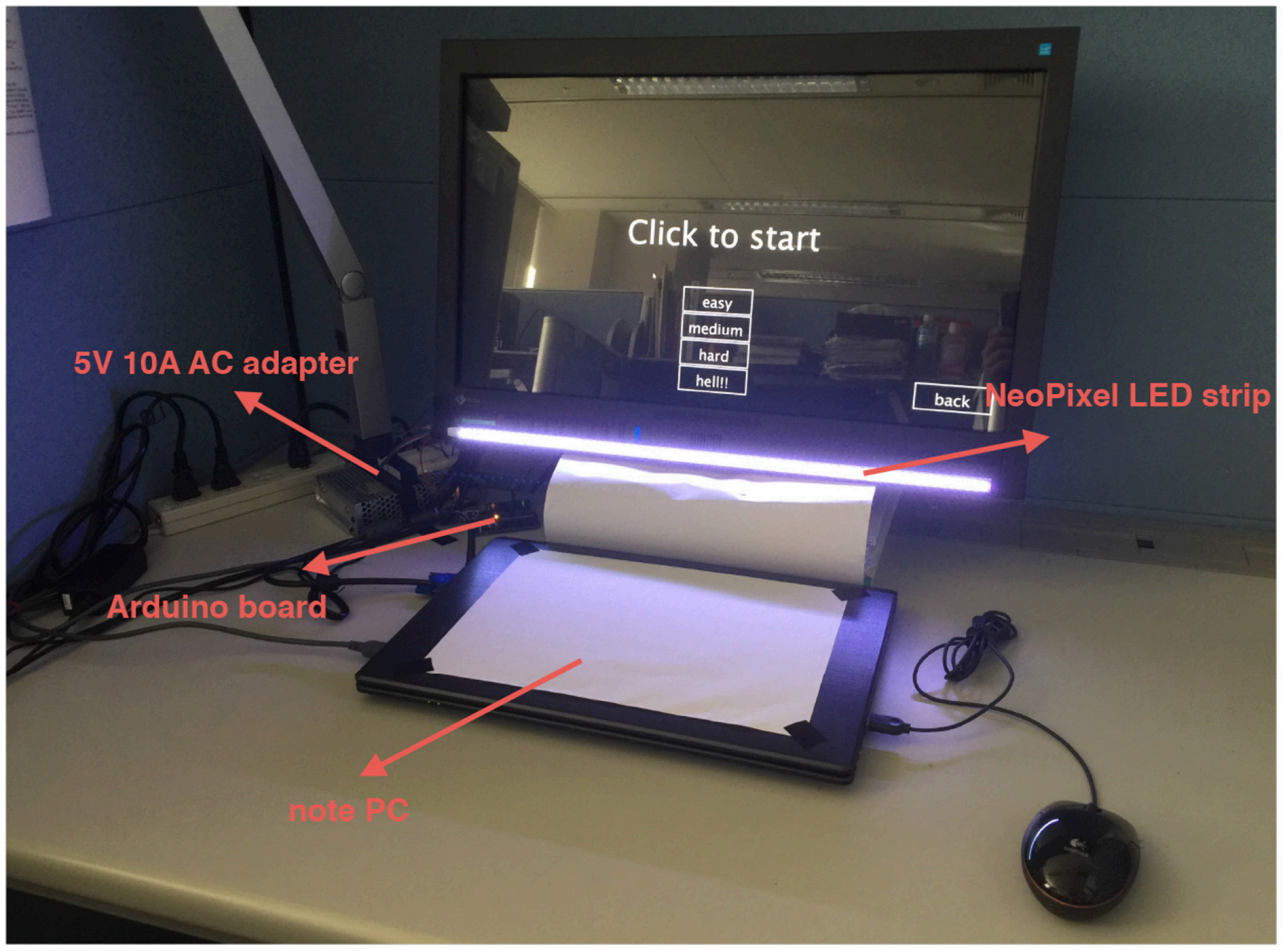
Setting of experiment environment.

Figure 3 illustrates the setting of the experiment environment. Basically, a notebook PC was used to run the ping-pong game software developed in Processing. A monitor was connected to the notebook PC to display the game. During the experiment, the notebook PC's cover was kept closed, and the game was played via the monitor in full-screen mode. A NeoPixel LED strip was attached to the bottom side of the monitor. The LED strip was controlled by an Arduino UNO board and powered by a 5-V, 10-A AC adaptor. It needs to be clarified that the image in Figure 3 was taken in a dark environment for the purpose of showing the light effect clearly. The actual experiment was done in a bright environment.

#### 3.1.2. Design of Light Expressions

**Figure 4:**
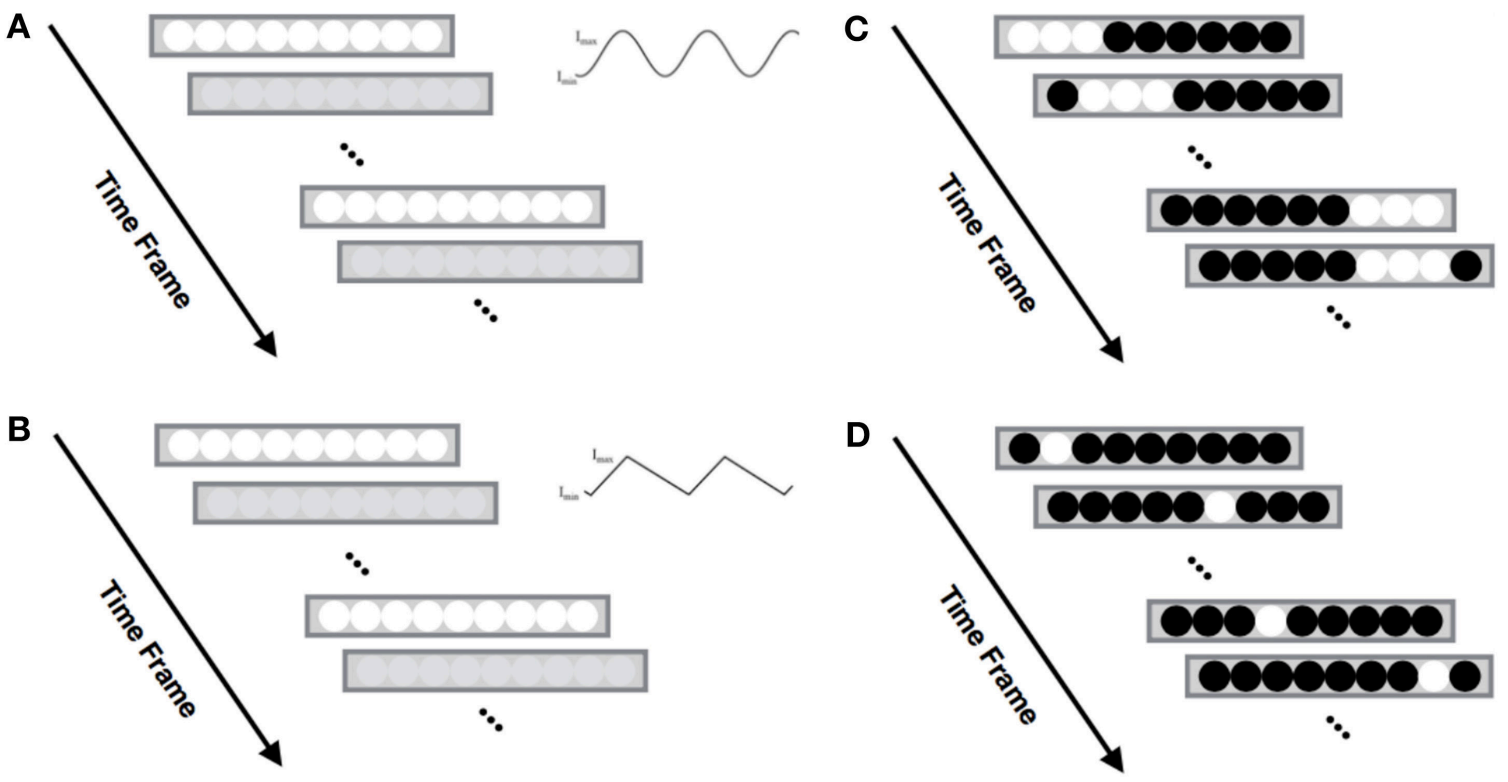
Set of light patterns we designed for ping pong game. **(A)** Sinusoidal waveform, **(B)** Triangle waveform, **(C)** *Swipe* animation, **(D)** *Random* animation.

On the basis of the defined animation space 1, we designed a set of four parameterized light animation patterns: sinusoidal, triangle, swipe, and random (see Figure 4). Patterns (Figures 4A,B) consist of two basic periodic waveforms, sinusoidal and triangle, and (Figures 4C,D) are patterns based on the whole LED strip. The parameters *I*_*min*_ and *I*_*max*_ are the minimum and maximum intensity values for the RGB color channels, and duty ratio *D* is the ratio of the rise time to period T. Table 1 demonstrates the set of light animations for each game event. Particularly, the light animation for the game-over event consists of both a sinusoidal pattern (first) and random pattern (after).

**Table 1:** Set of light animations for each game event.

**Light animation**	**Period (T, second)**	**Duty ratio (D)**	***I*_*min*_**	***I*_*max*_**	**Event**
Sinusoidal	2	–	RGB: 0, 0, 0	RGB: 255, 255, 255	Waiting for game to start
	0.2	–	RGB: 0,0,0	RGB: 255, 0, 0	game over
Triangle	0.6	60%	RGB: 0, 0, 0	RGB: 0, 255, 0	Ball hits racket
	0.6	60%	RGB: 0,0,0	RGB: 0, 0, 255	Ball hits wall
Swipe	–	–	RGB: 255, 255, 255	RGB: 255, 255, 255	Playing
Random	–	–	RGB: 100, 100, 100	RGB: 255, 255, 255	Game over

We tried to design the light animations to match with their corresponding events. For instance, we assumed that people would be calm when they were waiting for the game to start. Therefore, we chose a sinusoidal waveform with low intensity (2-s period) to match with this event. Oppositely, we presumed that people would be aroused and probably be upset and annoyed when they missed the ball (the goal was to bounce the ball by moving the racket). We thus used high intensity lights (0.2-s period) to match with this event.

#### 3.1.3. Procedure

We recruited 22 Japanese in total (nine males) for the experiment. Their ages ranged from 20 to 38 years old (*M* = 28.09, SD = 6.23). We designed two between-subject conditions: one with light animation and one without light animation.

Basically, the experiment was designed in two phases: a practice phase and a compete phase. In the practice phase, each participant practiced the game freely with access to all four difficulty levels. No time limit was given, so they were able to end the practice phase at any time when they felt comfortable with playing the game. In the compete phase, each participant selected one difficulty level only and played three rounds with regard to this difficulty level. His or her final score was decided as the highest score among the three rounds.

The participants were first welcomed by an experimenter and asked to sign some administrative documents. After this, the experimenter explained the ping pong game to the participants. They were asked to practice the game freely with no time limit before they were ready to “compete” with the others. When the participants thought that they had sufficient practice, they were then required to choose one difficulty mode and play three rounds in the same mode.

#### 3.1.4. Measurement

We carefully designed our post-questionnaire on the basis of Hart and Staveland (1988), Bartneck et al. (2009), and Lohse et al. (2014). It consisted of 22/21 items in total regarding the two experiment conditions with/without LED light animation. The questionnaire used for the with-LED condition contained one more question, “Did you notice the LED light animation?,” to check for manipulation. Each questionnaire had three types of items: yes/no questions, 7-point Likert-scale questions, and open questions, where 7-point Likert-scale questions were used the most (18 items). The yes/no questions were used to check for manipulation and for participant-related information.

In particular, the 7-point Likert-scale questions were designed with four main categories: Participant's

- **Perception of game**—included questions such as “Do you like this game?” and “Have you enjoyed playing the game?”
- **Perception of computer**—included questions such as “How much fun did you have using this computer?,” “Do you feel close to the computer?", and “Do you think the computer is alive?"
- **Rate of his or her gaming performance**—included questions such as “Do you think you are good at this game?” and “How competitive do you think your score is compared with others?”
- **Rate of his or her perceived workload**—included questions such as “Did you feel tired when playing the game?” and “Did you feel pressure during the game?”

### 3.2. Results

We checked if the participants were familiar with the game. Half of the participants (11 out of 22) answered that they had played similar games before the experiment. However, this would not affect the experiment results as we asked the participants to freely practice the game with no time limit before the formal test. We also checked if the participants perceived the game to be difficult by using a Mann-Whitney U test. No significant difference was found between the two experiment conditions with/without LED light animation (with light animation: 5.45; without light animation: 5; *Z* = 0.37; n.s.). Besides, we checked if the selected difficulty level of game impacted the results. Most of the participants chose easy and medium mode and only 3 of them chose hard and hell mode. No evidence was found that selected difficulty level affected the experiment results. In addition, no significant difference was found between the two conditions with regard to average practice time (with light animation: 1 m 46.5 s; without light animation: 1 m 39.5 s; *Z* = 0.24; n.s.).

Table 2 summarizes the evaluation results. The participants in the with-light-animation condition liked playing the game significantly more than those in the without-light-animation condition. They also liked and anthropomorphized the computer more. However, there was no significant difference between the two experiment conditions in terms of subjectively rated performance and perceived workload. Therefore, there is no evidence suggesting that using light animation would affect the participants' subjective ratings of their gaming performance and cause them extra frustration and stress. The final score also shows no significant difference between the two conditions.

**Table 2:** Summary of evaluation results.

**Category**	**Sub-category**	**With light animation**	**Without light animation**	**Z**	**p-value**	**η^2^**
		**Median**	**Median**			
Perception of game	–	5.67	4.33	3.21	*p* < 0.001	0.47
Perception of computer	Likeness	5	3.25	3.26	*p* < 0.001	0.48
	Anthropomorphism	2.83	2	2.69	*p* < 0.01	0.33
Rate of gaming performance	Capability	3	2	0.84	n.s.	0.03
	Competitiveness	2.5	2.5	−0.23	n.s.	0.00
Rate of perceived workload	frustration	2.5	2	1.61	n.s.	0.12
	Stress	5	5	−0.07	n.s.	0.00
Final score	–	60	30	1.42	n.s.	0.09

### 3.3. Discussion

The results suggest that using light animations to improve a user's experience with using a computer is promising. Specifically, we show that light animations can have a positive effect on a user's perception of a computer. This method is simple and therefore can be readily used to currently-in-use devices, e.g., computers, as lighting components such as LEDs can be easily embedded to them.

Our results reveal the interesting phenomenon that people anthropomorphize devices more when they include light animations. Although the link between light animation and anthropomorphism is unclear, we envision that expressive lights can be applied to intelligent devices and machines that require affective interaction abilities. This would not only improve the user experience with such devices but also facilitate in achieving more harmonious interactions with people.

In this work, we mounted an LED strip to the front-bottom of a monitor on the basis of previous studies on peripheral cognition technology (Yamada et al., 2013). Our results indicate that setting an LED strip in such a way may not have a negative effect on a user's task performance and lead to an increase in workload. Thus, such a setting is recommended. However, other settings such as the positions and number of LED strips used to display light animations need to be further explored.

## 4. Study 2

### 4.1. Design of Light Expressions

Previous research (Elliot and Maier, 2007) claimed that color meanings can be grounded in two sources: learned associations that develop from repeated pairings of colors with particular concepts or experiences and biologically based proclivities to respond to particular colors in particular ways in particular situations. For instance, a specific red-danger association can be generated from experiences with regard to (life-threatening) situations such as viewing blood, an angry face, traffic lights, and/or warning signals and sirens (Elliot and Maier, 2014). Similarly, green can be associated with positive meanings, e.g., approach and pleasure, due to experiences with green traffic lights and the general image of being the color of the natural. Besides, Terada et al. (2012) studied color and dynamic parameters for representing emotions. They found that a rectangular waveform with a high frequency represents intense emotions, while a sinusoidal waveform with a low frequency represents weak (low intensity) emotions.

In this study, we chose two colors: green and red. They are able to produce opposite effects on human psychological functioning. In general, green can be associated with positive perception, while red can be associated with negative perception. Further, we combined a sinusoidal waveform and a low frequency with green to enhance the effect of the color green. Similarly, we combined a rectangular waveform and a high frequency with red to enhance the effect of the color red. As a result, we design two light expressions: *GL* (green, low frequency, and sinusoidal waveform) and *RH* (red, high frequency, and rectangular waveform). Table 3 lists the two expressive lights.

**Table 3:** Set of light animations for each game event.

**Light animation**	**Period (T, second)**	**Duty ratio (D)**	***I*_*min*_**	***I*_*max*_**	**Expected effect**
Sinusoidal	1	−	RGB: 0, 0, 0	RGB: 0, 255, 0	Induce positive Perception
rectangle	0.2	50%	RGB: 0, 0, 0	RGB: 255, 0, 0	Induce negative perception

### 4.2. Method: Ultimatum Game

#### 4.2.1. Experiment Design

There are two players in the Ultimatum game (Sanfey et al., 2003; Torta et al., 2013): a proposer and a receiver. They are given the opportunity to split an amount of money. The proposer makes an offer as to how this money should be divided. The receiver can choose to either accept or reject this offer. If the receiver accepts the offer, the money is split according to the proposal. If the receiver rejects, neither player receives any money. In either case, the game is over.

Conventional human decision-making theories suggest that most humans, as rational agents, would accept any non-zero offer to maximize the benefit. However, recent research has revealed that people tend to reject lower offers (*p* < 30% of the amount of money) (Sanfey et al., 2003; Torta et al., 2013). It appears that people perceive such offers as unfair, and the negative emotions evoked by the unfair offers can lead people to sacrifice financial gain in order to punish their partner. In this work, we applied the Ultimatum game to observe the behavior of human players toward non-human—i.e., computer—opponents. Specifically, we wanted to see how their tolerance to unfair offers changed when the computer showed different light animations.

#### 4.2.2. Procedure

Twenty Japanese individuals (10 males, 10 females) ranging from 21 to 38 years old (*M* = 28.9, SD = 4.66) were recruited for the experiment. The experiment had a 3 (Light Animation: GL vs. RH vs. without light animation) × 4 (Offer: 50%50% vs. 70%30% vs. 80%20% vs. 90%10%) within-participant design.

The experimenter welcomed the participants, explained the game, and gave instructions. Each participant completed a total of 36 rounds (each combination of the levels of the two factors was repeatedly shown three times within the 36 rounds). Since the rounds were presented randomly, there was almost no learning effect. The computer showed a black screen for four seconds after each round, and the participants were asked to treat each round as an independent game. The total amount of money was set to 1,000 Japanese yen, which is roughly equal to 10 US dollars.

#### 4.2.3. Results

We checked if the participants were familiar with the game. None of the participants answered that they had played similar games before the experiment.

**Figure 5:**
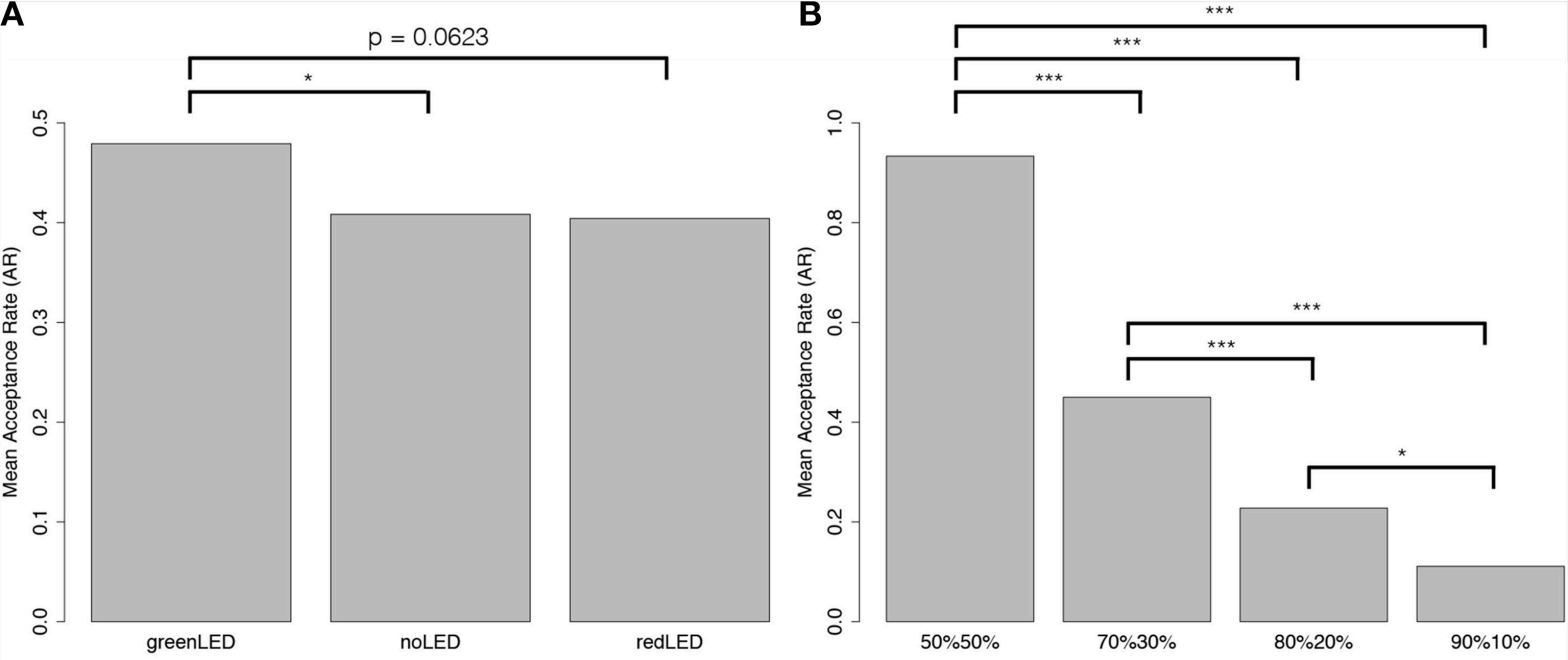
Results of the Ultimatum game. **(A)** Offer, **(B)** Light animation. ^*^p < 0.05; ^***^p < 0.001.

An aligned rank transform (ART) for nonparametric factorial data analysis was conducted to determine the effect of two independent factors (light animation vs. offer) on the acceptance rate as a dependent factor. Significant difference was found in the main effect of the type of offer [*F*_(3, 209)_ = 128.25, *p* < 0.001, $\eta_{p}^{2}=0.57$]; see Figure 5B. This is expected, as previous studies have indicated that the lower the offer, the lower the acceptance rate (Torta et al., 2013). Significant difference was also found in the main effect of type of light animation [*F*_(2, 209)_ = 4.57, *p* < 0.05, $\eta_{p}^{2}=0.02$]; see Figure 5A. The Tukey least-squares-means test showed that participants accepted offers made when the computer displayed GL more than when it displayed RH (*p* = 0.0623, marginally significant) or no light animation (*p* < 0.05), but no significant difference was found between when the computer displayed RH and no light animation. No significant difference was found in the interaction effect.

### 4.3. Method: Give-Some Game

#### 4.3.1. Experiment Design

In the Give-Some game, each participant is given four tokens, each worth a certain amount of money to the participant if he or she keeps it, but more if given to the partner. Therefore, maximal cooperation and communal gain occur if each participant gives all four tokens to his or her partner, while maximal individual gain accrues to someone who keeps all four tokens to him- or herself and receives all four tokens from his or her partner (DeSteno et al., 2012).

In this work, we applied the Give-Some game to observe the behavior of human players toward non-human opponents (i.e., computers). We adapted the original game to our study. Specifically, maximal cooperation (i.e., trustworthy) behavior is observed if a participant gives all four tokens to the computer, while maximal selfish (i.e., untrustworthy) behavior is observed if a participant keeps all four tokens to his- or herself (DeSteno et al., 2012).

#### 4.3.2. Procedure

The same twenty Japanese who were recruited for the Ultimatum game experiment also participated in this experiment (after a short break). The experiment had a 3 (Light Animation: GL vs. RH vs. without light animation) within-participant design.

Each participant completed three rounds (each level of the light animation factor), and the rounds were presented randomly. The computer showed a black screen for four seconds after each round, and the participants were asked to treat each round as an independent game. Each token was set to be worth 100 Japanese yen, which is roughly equal to 1 US dollar.

We also designed a post-game questionnaire to investigate the subjective perception of the participants on the LED light animations. The questionnaire contained both yes/no questions and open questions. The yes/no questions, such as “Have you played this kind of games before?,” were mainly used to find out about the manipulation. The open questions include questions such as “How did you think of the computer when it showed green/red light?” and “Please write down your comments on this game.”

#### 4.3.3. Results

We checked if the participants were familiar with the game. None of the participants answered that they had played similar games before the experiment.

**Figure 6:**
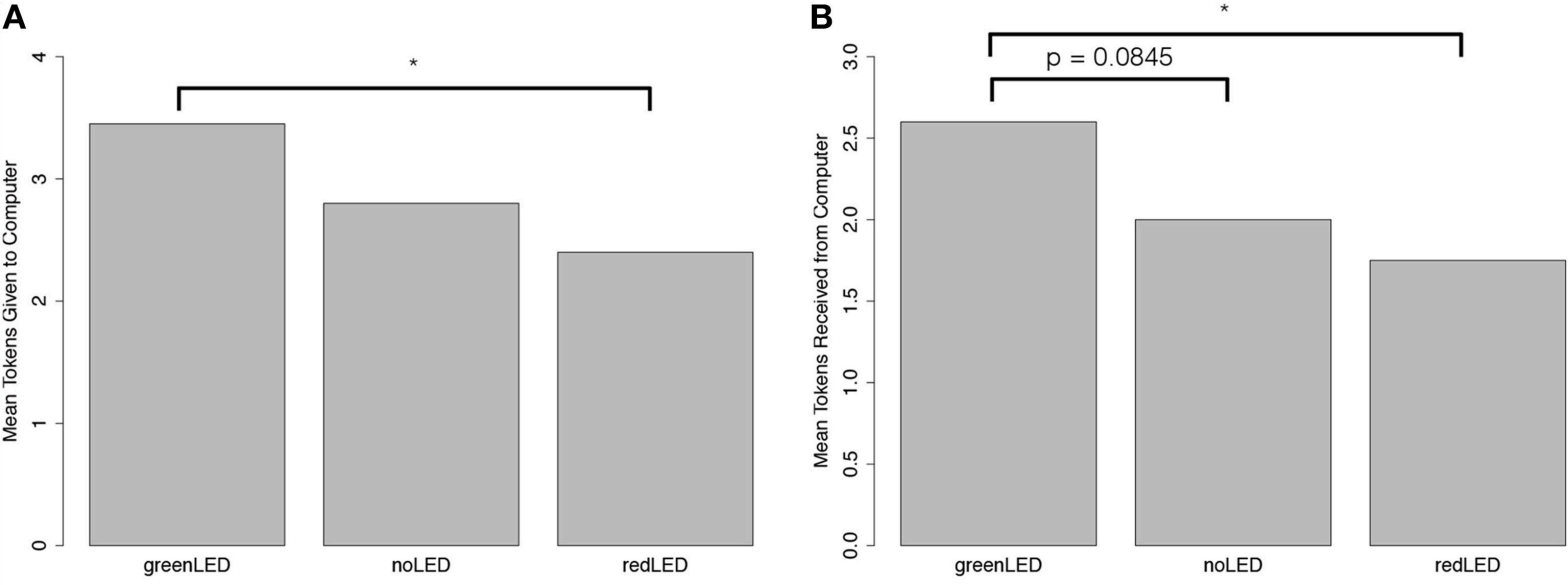
Results of Give-Some game. **(A)** Mean tokens given to computer. **(B)** Mean tokens expected from computer. ^*^p < 0.05.

Non-parametric Friedman tests were conducted to determine the effect of the independent factors (light animation) on the two dependent factors (number of tokens given to the computer and number of tokens expected from the computer). Light animation had a significant effect on the number of tokens given to the computer (Chi-square = 16.21, *p* < 0.001, $\eta_{p}^{2}=0.11$); see Figure 6A. Wilcoxon signed-rank test with Holm's correction posthoc analysis showed that participants gave tokens to the computer when it displayed GL more than when it displayed RH (*p* < 0.05). We also found that light animation had a significant effect on the number of tokens expected from the computer (Chi-square = 7.32, *p* < 0.05, $\eta_{p}^{2}=0.07$); see Figure 6B. Wilcoxon signed-rank test with Holm's correction posthoc analysis also showed that the participants expected more tokens from the computer when it displayed GL than when it displayed RH (*p* < 0.05) or no light animation (*p* = 0.0845, marginally significant).

### 4.4. Post-game Questionnaire

Analysis of the post-game questionnaire showed that 17 out of 20 participants used contrasting descriptions for the two light animations, as listed in Table 4. Two participants said that they did not notice any difference and one indicated that the LED lights reminded him of gambling machines.

**Table 4:** List of adjectives used by participants to describe the light animations.

	**Green and low intensive**	**Red and high intensive**	**No light animation**
Description	Friendly (12), calm (9), gentle (6), smiling (1), beautiful (1), kind (5), alive (2)	Angry (14), oppressive (6), feeling of tension (2), warning (7), challenging (3), dangerous (3)	Normal (20)

*Numbers in parenthesis indicate number of participants who gave comments*.

### 4.5. Discussion

The results indicate that the participants anthropomorphized the computer and treated it as a social agent, although such a process may be unconscious. They used adjectives such as “friendly” and “angry” to describe the computer, where such descriptions are generally applied to humans. Interestingly, the participants were more willing to accept unfair offers (the Ultimatum game) and were more cooperative (the Give-Some game) when they formed positive impressions of the computer compared with negative ones. This shows strong evidence that expressive lights affected the participants' perception of the computer and, more importantly, influenced their decision-making toward the computer's offers.

## 5. General Discussion

The two studies reveal significant results regarding the effect of expressive lights on human perception and behavior. The results of the PingPong game experiment showed that the participants preferred the computer displaying event-driven light animations and anthropomorphized it more, although the light animations were not exactly designed to express affect. However, it was not clear whether and how such a variation in perception can actually change people's attitude and behavior toward the computer. Therefore, we performed two more experiments, the Ultimatum game and the Give-Some game (both widely used in many fields to study human decision-making mechanisms). We found that the participants had positive impressions of the computer when it displayed green and low intensity light animation but negative impressions when it displayed red and high intensity light animation. Specifically, the participants were more willing to accept unfair offers (the Ultimatum game) and were more cooperative (the Give-Some game) when they formed positive impressions of the computer compared with negative ones. Our analysis of the post-experiment questionnaires confirmed these findings, as indicated by the participants using positive adjectives such as “friendly” and “kind” to describe the computer when it displayed GL and negative adjectives such as “angry” and “oppressive” when it displayed RH. We conclude that expressive lights can be an effective modality that facilitates human-machine interaction.

Our results suggest that color has strong effect on people's perception and decision-making. Basically, color psychologists have being focusing on red and green since such colors have been considered to be special and have positive links in the natural realm. Elliot and Maier (2007) claimed that each color activities associations that contain psychologically relevant messages. Therefore, viewing a color can influence psychological functioning and foster motivational and behavioral process such as approach and avoidance. Red can be associated with danger and anger and further induce avoidance-like behavior in people, whereas green carries positive meanings and can further induce approach-like behaviors.

We summarize our findings as three general design implications:

- Event-driven light displays can increase people's experience of using a computer.
- People have positive impressions of and further act approach-like behavior to a computer when it shows green light (combined with a sinusoidal waveform and a low frequency).
- People have negative impressions of and further act avoidance-like behavior to a computer when it shows red light (combined with a rectangular waveform and a high frequency).

It should be noted, however, that such design implications, especially II and III, may depend on people's attribution of a computer. Although most participants in our experiments attributed agency to the computer when it showed light displays, a few of them did not perceive any differences. Effect sizes of significant results of study 1 are fairly large, suggesting that event-driven light animations can have strong and positive influence to people's experience of using a computer. However, effect sizes of significant results of study 2 are overall small, indicating that effects of light animations on people's decision-making may not be very strong and reliable. Therefore, future research and applications may take user's personality into account.

The findings can open up possibilities for the design of ambient light systems for various applications. Previous studies with regard to ambient light displays mainly focused on informing users of certain information, e.g., progress on tasks and notifications. In this work, we show that LED lights can be applied to influence people's perception and decision-making. This effect can be used to support the design of ambient light systems for different applications. An ambient light system mounted to a computer monitor can be effective in scenarios such as entertainment, education, and social interaction. For instance, light animations can be designed to improve gaming experiences and make the computer more attractive, help people to relax and concentrate on study-related tasks, support in communicating social cues, and influence people's behavior and decision-making.

To achieve such goals, it is thus important to design appropriate expressive lights for specific applications and purposes. We, in this work, mainly focused on the exploration of the effects that expressive lights have on people. Therefore, we pre-designed our light animations on the basis of the findings from previous work (Terada et al., 2012; Baraka and Veloso, 2018). We did not intend to treat the parameters, e.g., color, waveform, and intensity, as independent factors as this would unnecessarily increase the complexity of our studies and make experimental results hard to explain. However, there may inevitably be concerns about an interaction effect among the factors, making it difficult to understand if the effects of expressive lights are to be attributed more to a particular parameter. Thus, future work can investigate the effects of individual parameters to contribute to better understanding of the design of effective light expressions.

Besides the design of expressive lights, further exploration into other factors, e.g., where and how to mount the LEDs, can reveal interesting findings and practical design implications of ambient light systems. In this work, we mounted a programmable LED strip to the front-bottom of a computer monitor as we assumed that such a place was within a user's peripheral visual field and thus would not distract him or her. However, other places of the monitor and more LED strips can be investigated as well. Such findings can be obtained to support the general design of ambient light systems for various other devices such as smart home devices and robots.

Compared with on-screen displays, ambient light systems have advantages in that they communicate in the periphery of people's attention without distracting them from their primary task. Previous work (Müller et al., 2015) also suggested that people would prefer ambient light systems over on-screen displays. Matviienko et al. (2015) described four information classes, progress, status, spatial, and notification, that an ambient light system can convey. However, our findings show evidence that expressive light animations can be designed to achieve more functionalities, e.g., influencing people's perception and decision-making. Therefore, future work should explore more application scenarios for the use of ambient light systems.

## Ethics Statement

All studies were carried out in accordance with the recommendations of the Ethical Guidelines for Medical and Health Research Involving Human Subjects provided by the Ministry of Education, Culture, Sports, Science and Technology and Ministry of Health, Labor, and Welfare in Japan with written informed consent from all participants. All participants gave written informed consent in accordance with the Declaration of Helsinki. The protocol was approved by the ethics committee of the National Institute of Informatics.

## Author Contributions

SS implementation of the study, drafting the work. SS and SY conception of the paper, revising the draft.

### Conflict of Interest Statement

The authors declare that the research was conducted in the absence of any commercial or financial relationships that could be construed as a potential conflict of interest.
